# Distinct Parietal and Temporal Pathways to the Homologues of Broca's Area in the Monkey

**DOI:** 10.1371/journal.pbio.1000170

**Published:** 2009-08-11

**Authors:** Michael Petrides, Deepak N. Pandya

**Affiliations:** 1 Montreal Neurological Institute, McGill University, Montreal, Quebec, Canada; 2 Department of Psychology, McGill University, Montreal, Quebec, Canada; 3 Department of Anatomy, Boston University School of Medicine, Boston, Massachusetts, United States of America; 4 Department of Neurology, Beth Israel Deaconess Medical Center, Boston, Massachusetts, United States of America; 5 ENR Memorial Veterans Administration Hospital, Bedford, Massachusetts, United States of America; NIMH-NIH, United States of America

## Abstract

The homologues of the two distinct architectonic areas 44 and 45 that constitute the anterior language zone (Broca's region) in the human ventrolateral frontal lobe were recently established in the macaque monkey. Although we know that the inferior parietal lobule and the lateral temporal cortical region project to the ventrolateral frontal cortex, we do not know which of the several cortical areas found in those regions project to the homologues of Broca's region in the macaque monkey and by means of which white matter pathways. We have used the autoradiographic method, which permits the establishment of the cortical area from which axons originate (i.e., the site of injection), the precise course of the axons in the white matter, and their termination within particular cortical areas, to examine the parietal and temporal connections to area 44 and the two subdivisions of area 45 (i.e., areas 45A and 45B). The results demonstrated a *ventral* temporo-frontal stream of fibers that originate from various auditory, multisensory, and visual association cortical areas in the intermediate superolateral temporal region. These axons course via the extreme capsule and target most strongly area 45 with a more modest termination in area 44. By contrast, a *dorsal stream* of axons that originate from various cortical areas in the inferior parietal lobule and the adjacent caudal superior temporal sulcus was found to target both areas 44 and 45. These axons course in the superior longitudinal fasciculus, with some axons originating from the ventral inferior parietal lobule and the adjacent superior temporal sulcus arching and forming a simple arcuate fasciculus. The cortex of the most rostral part of the inferior parietal lobule is preferentially linked with the ventral premotor cortex (ventral area 6) that controls the orofacial musculature. The cortex of the intermediate part of the inferior parietal lobule is linked with both areas 44 and 45. These findings demonstrate the posterior parietal and temporal connections of the ventrolateral frontal areas, which, in the left hemisphere of the human brain, were adapted for various aspects of language production. These precursor circuits that are found in the nonlinguistic, nonhuman, primate brain also exist in the human brain. The possible reasons why these areas were adapted for language use in the human brain are discussed. The results throw new light on the prelinguistic precursor circuitry of Broca's region and help understand functional interactions between Broca's ventrolateral frontal region and posterior parietal and temporal association areas.

## Abstract 2

An unprecedented detailed analysis of ventrolateral frontal cortical circuitry in Broca's area of the non-human primate brain clarifies the functional pathways permitting interaction between posterior cortical areas and the anterior language zone, providing important clues about the evolution of language.

## Author Summary

Two distinct cortical areas in the frontal lobe of the human brain, known as Broca's region, are involved with language production. This region has also been shown to exist in nonhuman primates. In this study, we explored the precise neural connectivity of Broca's region in macaque monkeys using the autoradiographic method to achieve a level of detail impossible in the human brain. We identified two major streams of connections feeding into Broca's area: a ventral stream from the temporal region, which includes areas processing auditory, multisensory, and visual information and a dorsal stream originating from the inferior parietal lobule and the adjacent superior temporal sulcus. Our detailed connectivity analysis illuminates the pathways via which posterior cortical areas can interact functionally with Broca's region, in addition to contributing to an understanding of the evolution of language. We suggest that a fundamental function of Broca's region is to retrieve information in a controlled strategic way from posterior cortical regions and to translate this information into action. This fundamental function was adapted during evolution of the left hemisphere of the human brain to serve language.

## Introduction

**Figure 1:**
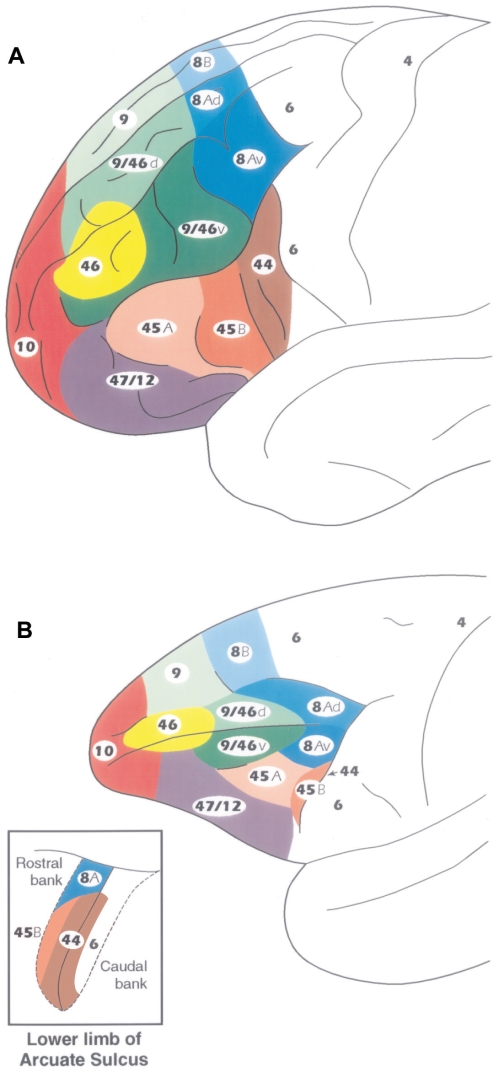
Architectonic map of the human and macaque monkey prefrontal cortex. (A) Lateral surface of the human prefrontal cortex. (B) Lateral surface of the macaque monkey prefrontal cortex according to Petrides and Pandya [8],[9]. The inset in (B) shows the opened anterior and posterior banks of the lower (inferior) limb of the arcuate sulcus of the monkey to illustrate the position of area 44 that lies in the fundus of this part of the sulcus. Area 45 extends anterior to it as far as the IPD on the surface of the inferior frontal convexity. The caudal subdivision of area 45 (i.e., area 45B) lies immediately in front of area 44 on the anterior bank of the sulcus and extends as far as the lip of the sulcus. Area 45A occupies the dorsal part of the inferior frontal convexity and is succeeded ventrally by area 47/12.

In the ventrolateral frontal lobe of the left hemisphere of the human brain, two distinct architectonic areas, areas 44 and 45, are involved with various aspects of language production and are considered to constitute the anterior language zone, which is also known as Broca's region (Figure 1A) [1]. Electrical stimulation of Broca's region during brain surgery leads to interference with speech production (e.g., [2]–[4]). Broca's region lies immediately anterior to the ventral part of the precentral gyrus, which, in both the human and nonhuman primate brains, is involved with the motor control of the orofacial musculature [2],[5],[6]. In the human brain, architectonic area 44 lies immediately anterior to the ventral precentral gyrus (i.e., in front of premotor area 6) and occupies the pars opercularis of the inferior frontal gyrus. It is succeeded, rostrally, by area 45 on the pars triangularis of the inferior frontal gyrus (Figure 1A) [1],[7]–[9]. While the primary motor cortex (area 4) and the ventral premotor cortex (area 6) have been consistently identified in the ventral part of the precentral region of the macaque monkey [10]–[12], there has been considerable confusion with the identification of areas 44 and 45. In the classic map of the macaque monkey by Walker [13], which has been adopted with minor modifications by most investigators of the monkey brain, area 44 was not identified and a narrow strip of cortex along the anterior bank of the inferior branch of the arcuate sulcus was labeled as area 45, although Walker repeatedly stated that he could not be certain whether it corresponded to area 45 of the human brain because he had not compared its architecture with that of the human (see [9] for discussion of this issue). Confusion also arose because the dorsal part of Walker's area 45 (i.e., the part immediately ventral to area 8) in the macaque monkey has been considered a visual oculomotor area by some investigators (e.g., [14]) which, clearly, would not be the case for an area that might be the homologue of a language zone.

The above confusing state of affairs led us to reexamine systematically the architecture of the frontal cortex of the macaque monkey in comparison with that of the human brain [8],[9]. This research demonstrated the following facts. First, a dysgranular area lying just anterior to the ventral premotor cortex (area 6) could be identified in the macaque monkey in the depth of the ventral part of the inferior branch of the arcuate sulcus, and this area had the architectonic characteristics of human area 44 (Figures 1B and S1). Furthermore, a combined anatomical–physiological study demonstrated that the neurons in the newly identified area 44 of the macaque monkey were involved with the orofacial musculature [15]. Dysgranular area 44 is succeeded, anteriorly, by area 45, which is a clearly granular cortex (Figure S1) with the architectonic characteristics of area 45 in the human brain: clusters of unusually large neurons in layer III, a well-developed layer IV, and moderate sized neurons in layer V (see [8],[9],[15]). We found that monkey area 45 *as defined by criteria comparable to those of the human brain* extends anteriorly as far as the infraprincipal dimple (IPD) and that it can be subdivided into a caudal (area 45B) and a rostral (area 45A) part (Figure 1B). Furthermore, monkey area 45 *when defined by the criteria of human area 45* is not related to oculomotor function as shown by a combined architectonic–microstimulation study that examined this issue [15]. The part of Walker's area 45 that had previously been linked to oculomotor function does not have the characteristics of human area 45 but rather those of caudal area 8 [15]. Ventral to the newly defined monkey area 45 lies area 12, which is comparable to a part of area 47 of the human brain and we have therefore labeled it as area 47/12 (Figure 1B). Area 47/12 is typical prefrontal cortex, i.e., it has a well developed layer IV, but the clusters of unusually large neurons in layer III that characterize area 45 are not observed in area 47/12 [9].

Several experimental anatomical studies of cortico-cortical connections had previously reported inputs to the ventrolateral frontal convexity of the monkey from the inferior parietal lobule [16]–[20], the superior temporal gyrus, and the cortex within the superior temporal sulcus [21]–[24], as well as from the adjacent dorsal inferotemporal cortex [25],[26]. These earlier studies, however, cannot provide precise information regarding the origin of axons within the many architectonic cortical areas that constitute the large inferior parietal and lateral temporal regions and the pathways by means of which these axons reach the newly identified homologues of Broca's region in the macaque monkey (i.e., areas 44, 45B, and 45A). The reason is that the terminations of axons in earlier studies were described in terms of older architectonic schemes that (a) did not identify area 44 and (b) included the cortex where the two subdivisions of area 45 lie as part of either area 8, or area 12, or area 46. In the few studies in which area 45 was identified, it was defined as a small strip of cortex along the anterior bank of the inferior branch of the arcuate sulcus following the criteria of Walker [13], which are not those used to define the macaque area 45 that is comparable with area 45 of the human brain (see [9],[15]). Thus, the terminations of the parieto-frontal and temporo-frontal fibers within the ventrolateral frontal region of the macaque as described in the older studies cannot be easily related to the homologues of the human Broca's region and, thus, inform our understanding of the connections of the human brain. It should be noted here that, although current diffusion tensor imaging methodology used in the human brain has been useful in providing an overall view of the large pathways that link the inferior parietal lobule and the superior lateral temporal region with the ventrolateral frontal region [27]–[31], it does not have the resolution to demonstrate the exact cortical origin of axons and their precise termination within particular target cortical areas. Thus, apart from stating that the inferior parietal lobule and the superior temporal region connect with the ventrolateral frontal region, such studies in the human brain have provided no information about the precise architectonic areas within the large posterior parietal and temporal cortical regions from which axons originate and the precise ventrolateral frontal architectonic areas within which these axons terminate. This information must be extrapolated from experimental anatomical studies in the macaque monkey in which appropriate anatomical methodology (e.g., autoradiography) can be used to establish such details.

Given the above considerations, the purpose of the present study was to examine, in the macaque monkey, the terminations within ventrolateral frontal areas 44, 45A, and 45B (i.e., the homologues of Broca's region) of axons originating within specific parietal and lateral temporal cortical areas and the pathways utilized to reach Broca's region. We used the autoradiographic method, which involves the injection of radioactively labeled isotopes in a particular cortical area of interest and provides an unambiguous demonstration of the precise course and terminations of axons that originate from the injected region [32]. Such information is critically needed because (a) it cannot be obtained in the human brain and (b) it can be used to guide hypotheses and provide plausible interpretations of connections in the human brain examined with diffusion tensor imaging (e.g., [27]–[31]) or physiological connectivity between cortical areas during baseline activation states (e.g., [33],[34]). Furthermore, such information on the circuitry of areas that, in the left hemisphere of the human brain, were adapted to serve certain aspects of language provides major insights into the prelinguistic antecedents of “language” areas and thus major insights into the evolution of language circuits.

## Results

**Figure 2:**
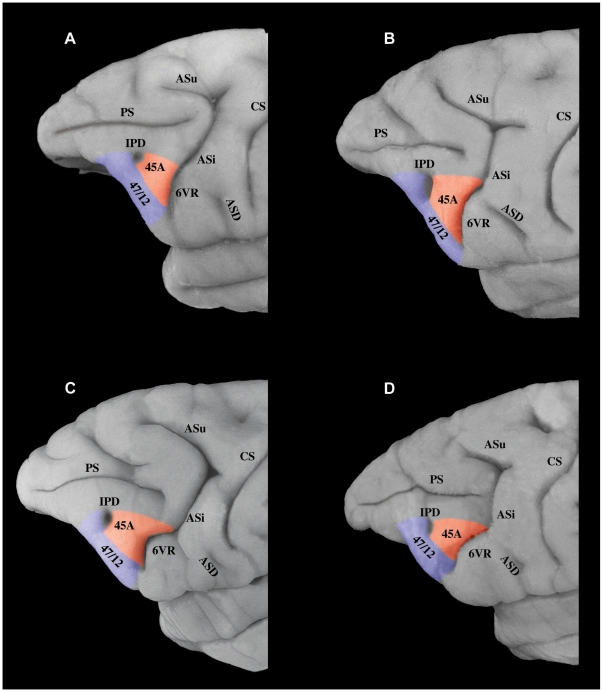
Variations in the morphology of the inferior limb of the arcuate sulcus. (A) Photograph to show a case in which the inferior limb of the arcuate sulcus (ASi) is a smooth extension of the rest of the sulcus. Nevertheless, note the clear downward turn of the most ventral part of the sulcus. (B–D) Photographs to show the ventral component of ASi which in many cases is, morphologically, a distinct branch. The homologues of Broca's area lie in relation to this ventral component of the ASi and the immediately adjacent dorsal part of ASi. Area 44 cannot be seen on the surface of the brain because it lies hidden in the depth of the sulcus. Area 45A extends anterior as far as the IPD on the surface of the inferior frontal convexity. Note that the IPD can be a small barely detectable dimple or a clear small sulcus.

**Figure 3:**
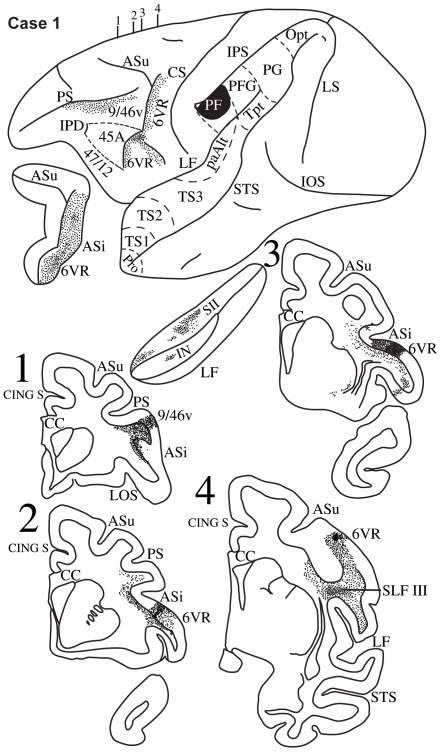
Case 1. Diagrammatic representation of the lateral surface of the cerebral hemisphere to show the location of the isotope injection in the most rostral part of the inferior parietal lobule (area PF) shown as solid black and the distribution of terminal label in the frontal lobe shown as dots. The upper (ASu) and inferior (ASi) branches of the arcuate sulcus and the lateral fissure have been opened up to show label in their banks. Coronal sections, at the levels indicated on the diagram of the lateral surface of the hemisphere, show the labeled pathways in the white matter. The architectonic areas of the inferior parietal lobule (PF, PFG, PG, Opt) and the superior temporal gyrus (TS1, TS2, TS3, paAlt, Tpt) have been marked on the lateral surface of the hemisphere. Pro refers to the temporal proisocortex.

The present study examined the origin, course, and terminations of axonal fiber systems connecting the various architectonic areas of the inferior parietal lobule and the lateral temporal region to the newly defined homologues of Broca's region, i.e., areas 44, 45B, and 45A, in the ventrolateral frontal cortex of the macaque monkey. Our architectonic studies have shown that area 44 lies in the fundus of the inferior branch of the arcuate sulcus, immediately in front of the rostral part of premotor area 6, and is succeeded on the anterior bank of this sulcus by area 45B, which extends onto the lip of the sulcus (Figure 1B). Area 45A extends rostrally as far as the IPD and is succeeded ventrally by area 47/12 [8],[9],[15]. Note that the IPD can be a barely detectable dimple (Figure 2A) or a small vertically oriented sulcus (Figure 2B–2D). Also note that the ventral-most part of the inferior branch of the arcuate sulcus, where the homologues of Broca's region lie, forms a distinct segment that makes a relatively sharp forward turn before continuing ventrally (Figure 2B–2D). It is important to note here that the architectonic areas within which the injections were placed (i.e., origins of the pathways) and the architectonic areas within which terminal label was observed in the ventrolateral frontal cortex was established in each individual case under microscopic examination. The sections shown in Figures 3–13 are drawings of actual histological sections examined and charted under darkfield microscopy to locate the labeled axons in the white matter and the axon terminations in the gray matter. Thus, in each individual case, terminal label was verified to be within the architectonic areas of interest on the basis of the criteria that we had established in earlier studies of the homologues of Broca's region [8],[9],[15] (Figure 1B). The posterior parietal cortical areas were defined according to the criteria of Pandya and Seltzer [35], the superior temporal gyrus according to Pandya and Sanides [36], and the inferotemporal cortex and the cortex of the superior temporal sulcus according to Seltzer and Pandya [37]. The architectonic areas of the parietal and superior temporal region from which axons originate and terminate within the homologues of Broca's region in the macaque monkey brain are also provided in the macaque monkey brain atlas by Paxinos and colleagues [38].

**Figure 4 pbio-1000170-g004:**
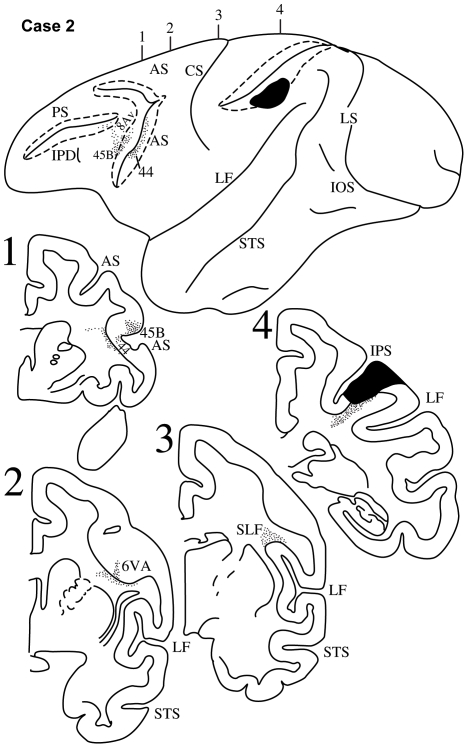
Case 2. Diagrammatic representation of the lateral surface of the cerebral hemisphere to show the location of the isotope injection in the upper part of the inferior parietal lobule involving the dorsal part of area PFG and part of the immediately adjacent lateral bank of the intraparietal sulcus (area LIP) shown as solid black and the distribution of terminal label in the frontal lobe shown as dots. Note the involvement of the lower bank of the intraparietal sulcus (dotted lines). The arcuate sulcus (AS) and the principal sulcus (PS) have also been opened up (dotted lines) to show label in their banks. Coronal sections, at the levels indicated on the diagram of the lateral surface of the hemisphere, show the labeled pathways in the white matter.

**Figure 5 pbio-1000170-g005:**
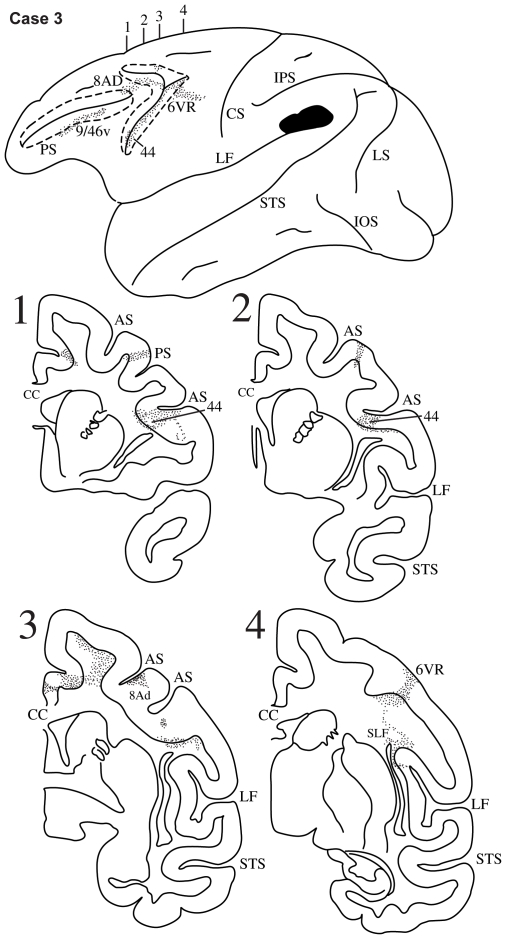
Case 3. Diagrammatic representation of the lateral surface of the cerebral hemisphere to show the location of the isotope injection in the lower part of the inferior parietal lobule involving the ventral part of area PFG just above the caudal end of the lateral fissure shown as solid black and the distribution of terminal label in the frontal lobe shown as dots. The arcuate sulcus (AS) and the principal sulcus (PS) have been opened up (dotted lines) to show label in their banks. Coronal sections, at the levels indicated on the diagram of the lateral surface of the hemisphere, show the labeled pathways in the white matter.

**Figure 6 pbio-1000170-g006:**
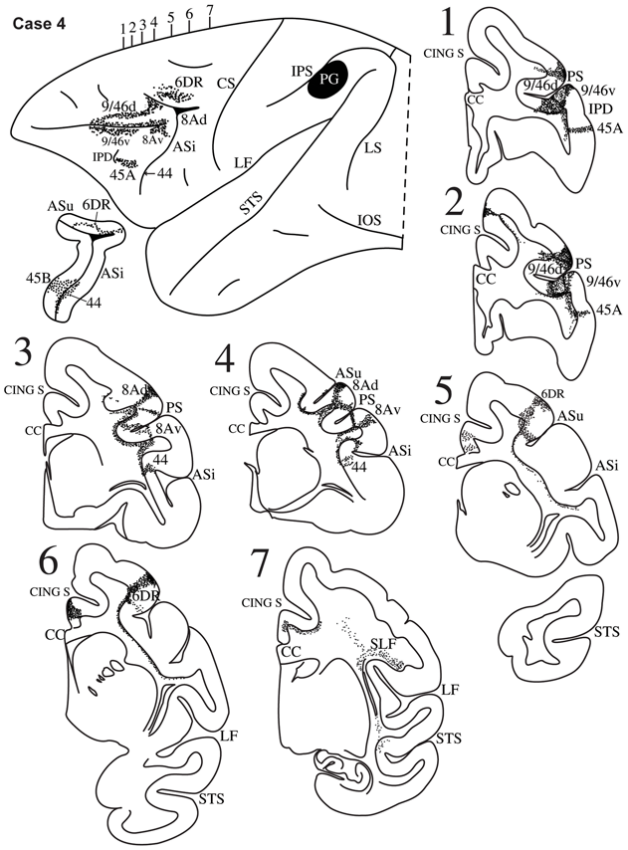
Case 4. Diagrammatic representation of the lateral surface of the cerebral hemisphere to show the location of the isotope injection in the upper part of the inferior parietal lobule involving the upper part of area PG shown as solid black and the distribution of terminal label in the frontal lobe shown as dots. The upper (ASu) and inferior (ASi) branches of the arcuate sulcus have been opened up to show label in their banks. Coronal sections, at the levels indicated on the diagram of the lateral surface of the hemisphere, show the labeled pathways in the white matter.

**Figure 7 pbio-1000170-g007:**
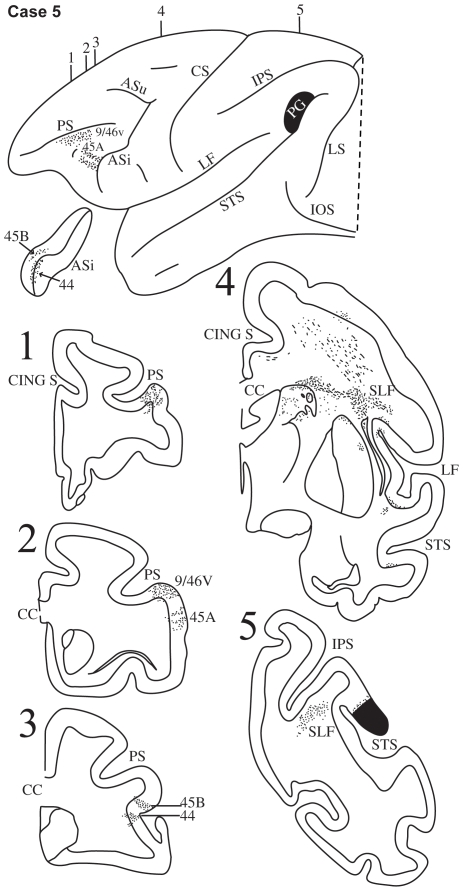
Case 5. Diagrammatic representation of the lateral surface of the cerebral hemisphere to show the location of the isotope injection in the lower part of the inferior parietal lobule involving the lower part of area PG and adjacent superior temporal sulcus shown as solid black and the distribution of terminal label in the frontal lobe shown as dots. The inferior ramus of the arcuate sulcus (ASi) has been opened up to show label in its banks. Coronal sections, at the levels indicated on the diagram of the lateral surface of the hemisphere, show the labeled pathways in the white matter.

**Figure 8 pbio-1000170-g008:**
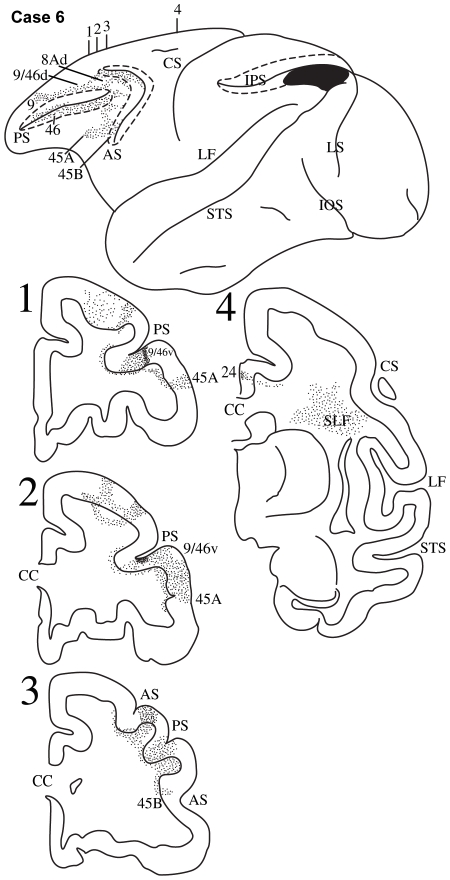
Case 6. Diagrammatic representation of the lateral surface of the cerebral hemisphere to show the location of the isotope injection in the most caudal part of the inferior parietal lobule involving the caudal part of area PG and area Opt shown as solid black and the distribution of terminal label in the frontal lobe shown as dots. The arcuate sulcus (AS) and the principal sulcus (PS) have been opened up to show label in their banks. Coronal sections, at the levels indicated on the diagram of the lateral surface of the hemisphere, show the labeled pathways in the white matter.

**Figure 9 pbio-1000170-g009:**
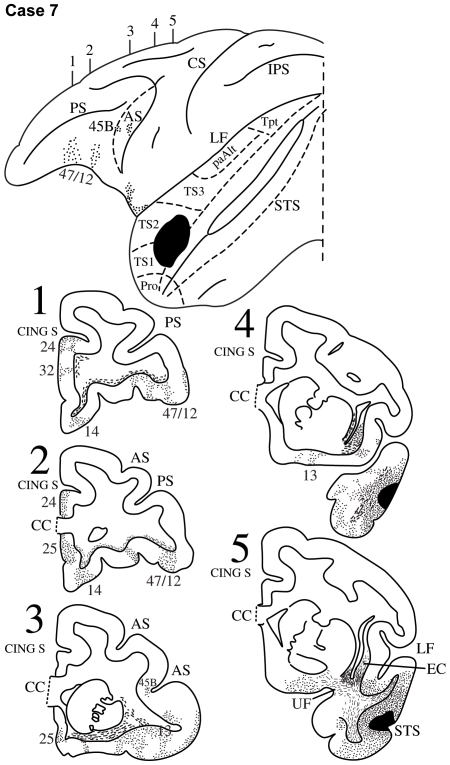
Case 7. Diagrammatic representation of the lateral surface of the cerebral hemisphere to show the location of the isotope injection in the rostral part of the superior temporal gyrus involving areas TS1 and TS2 shown as solid black and the distribution of terminal label in the frontal lobe shown as dots. The anterior bank of the inferior limb of the arcuate sulcus (AS) has been opened up (dotted lines) to show label in it. The superior temporal sulcus (STS) has been opened up (dotted lines) to show the extension of the injection in the upper bank of the sulcus. Coronal sections, at the levels indicated on the diagram of the lateral surface of the hemisphere, show the labeled pathways in the white matter.

**Figure 10 pbio-1000170-g010:**
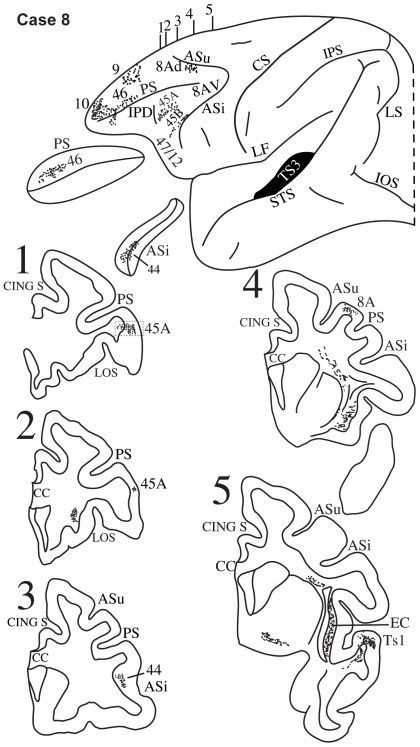
Case 8. Diagrammatic representation of the lateral surface of the cerebral hemisphere to show the location of the isotope injection in the intermediate part of the superior temporal gyrus involving area TS3 shown as solid black and the distribution of terminal label in the frontal lobe shown as dots. The sulcus principalis (PS) and the inferior limb of the arcuate sulcus (ASi) have been opened up to show label in their banks. Coronal sections, at the levels indicated on the diagram of the lateral surface of the hemisphere, show the labeled pathways in the white matter. A photomicrograph of the small part of the ventrolateral prefrontal cortex in section 1 is shown in Figure S2A.

**Figure 11 pbio-1000170-g011:**
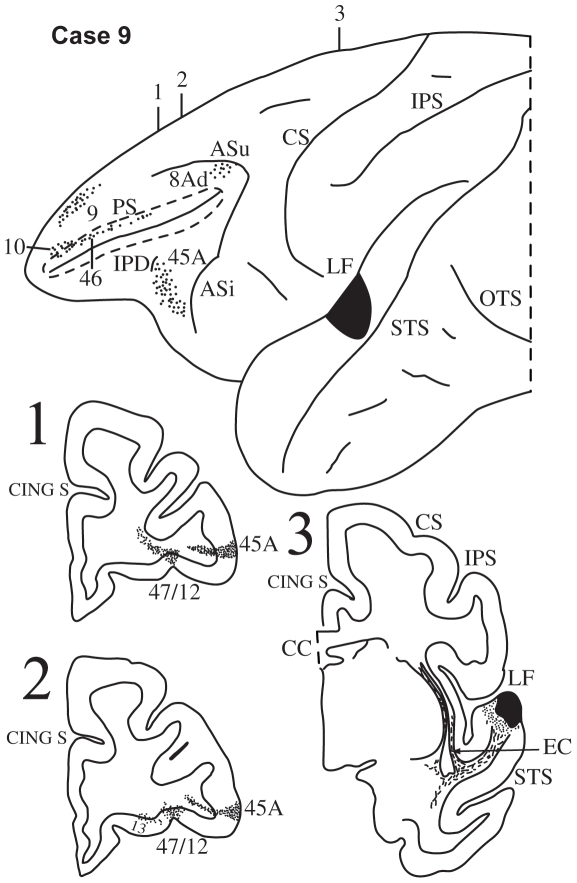
Case 9. Diagrammatic representation of the lateral surface of the cerebral hemisphere to show the location of the isotope injection in the upper part of the intermediate section of the superior temporal gyrus involving area paAlt shown as solid black and the distribution of terminal label in the frontal lobe shown as dots. The sulcus principalis (PS) has been opened up (dotted lines) to show label in its banks. Coronal sections, at the levels indicated on the diagram of the lateral surface of the hemisphere, show the labeled pathways in the white matter.

**Figure 12 pbio-1000170-g012:**
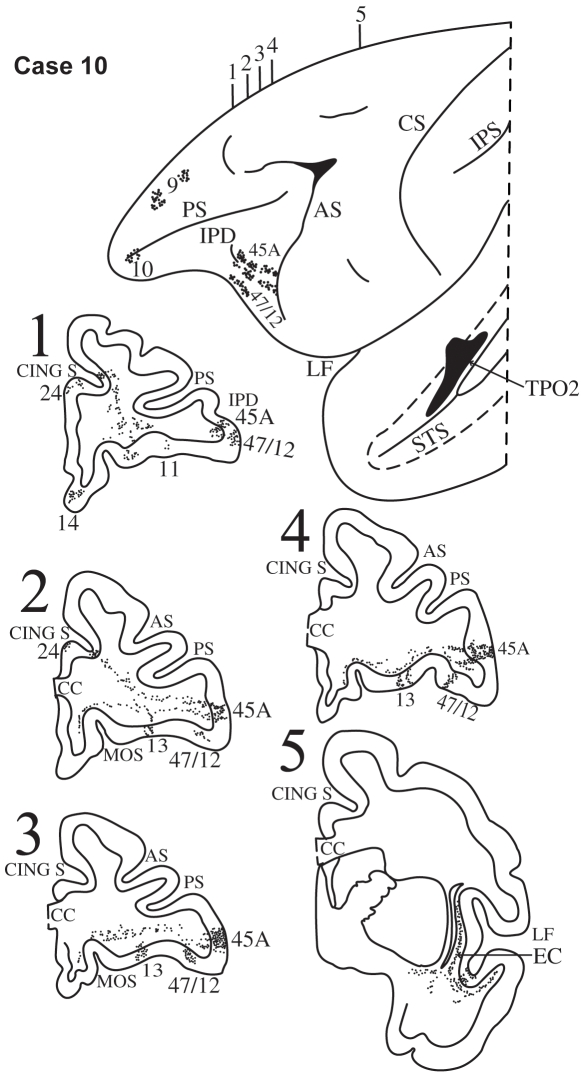
Case 10. Diagrammatic representation of the lateral surface of the cerebral hemisphere to show the location of the isotope injection in the upper bank of the superior temporal sulcus (dotted lines) involving area TPO2 shown as solid black and the distribution of terminal label in the frontal lobe shown as dots. Coronal sections, at the levels indicated on the diagram of the lateral surface of the hemisphere, show the labeled pathways in the white matter.

**Figure 13:**
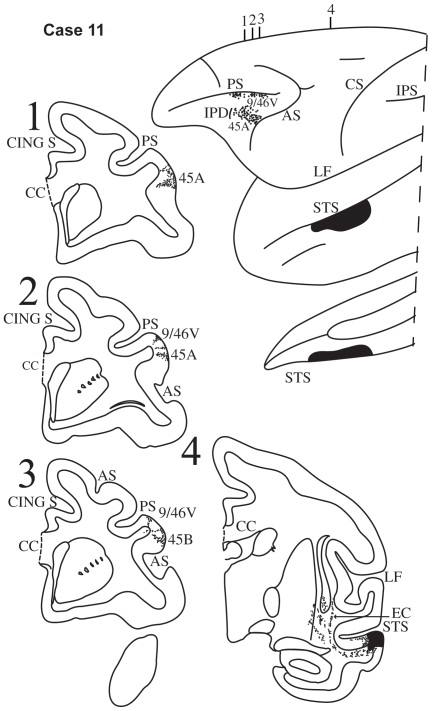
Case 11. Diagrammatic representation of the lateral surface of the cerebral hemisphere to show the location of the isotope injection in the upper part of the inferotemporal cortex close to the superior temporal sulcus involving the dorsal part of area TE and its extension into the adjacent ventral part of the sulcus (area TEm) shown as solid black and the distribution of terminal label in the frontal lobe shown as dots. Coronal sections, at the levels indicated on the diagram of the lateral surface of the hemisphere, show the labeled pathways in the white matter.

### Inferior Parietal Lobule

#### Case 1

In this case, the isotope injection was placed in the most rostral part of the inferior parietal lobule involving area PF (Figure 3). Labeled fibers were observed in the white matter of the parieto-frontal operculum in the region of the third branch of the superior longitudinal fasciculus (SLF III) (Figure 3, section 4). In the frontal lobe, terminal label was observed in the anterior part of the ventral premotor cortex (area 6) along the inferior branch of the arcuate sulcus. Labeled fibers also entered the caudal bank of the lower branch of the arcuate sulcus and terminated in the most rostral part of area 6 (Figure 3, sections 2, 3). Only minor terminal label was observed in the fundus of the sulcus in area 44. Further rostrally, terminal label was observed just ventral to the caudal part of the sulcus principalis in area 9/46v (Figure 3, section 1).

#### Case 2

The injection in this case involved the rostral part of the lower bank of the intraparietal sulcus extending onto the adjacent dorsal part of the inferior parietal lobule. The injection involved area POa in the intraparietal sulcus and the dorsal part of area PFG (Figure 4). Labeled fibers were observed in the white matter of the inferior parietal lobule continuing forward just above the circular sulcus of the insula occupying the third branch of the superior longitudinal fasciculus (SLF III) (Figure 4, section 3). In the frontal lobe, these labeled fibers terminated in the fundus of the ventral part of the inferior branch of the arcuate sulcus in dysgranular area 44 and in the anterior bank in granular area 45B (Figure 4, section 1). In the dorsolateral frontal cortex, some terminal label was observed in area 8Av, just above area 45B.

#### Case 3

The injection in this case involved the ventral part of the inferior parietal lobule just above the lateral fissure and thus occupied the ventral part of area PFG (Figure 5). Labeled fibers were observed in the adjacent white matter and just above the circular sulcus of the insula, i.e., in the third branch of the superior longitudinal fasciculus (SLF III) (Figure 5, section 4). These fibers continued rostrally in the white matter of the frontal lobe and terminated in the ventral part of the inferior branch of the arcuate sulcus. Some terminal label was observed in premotor cortex (area 6), but most label was in the fundus of the sulcus in area 44 (Figure 5, sections 1, 2). Terminal label was also observed along the ventral bank of the caudal sulcus principalis in area 9/46v. In the dorsolateral frontal cortex, label was observed in area 8AD.

#### Case 4

The isotope injection in this case was placed in the intermediate part of the inferior parietal lobule involving the dorsal part of area PG (Figure 6). Labeled fibers were observed in the white matter of the inferior parietal lobule and then just above the circular sulcus of the insula occupying the superior longitudinal fasciculus (SLF II) (Figure 6, section 7). In the ventrolateral frontal lobe, terminal label was observed in the depth of the ventral part of the inferior branch of the arcuate sulcus in area 44 (Figure 6, sections 3, 4), in area 45B in the anterior bank of the inferior branch of the arcuate sulcus, and in area 45A as far as the IPD (Figure 6, sections 1, 2). Label was also observed in the ventral bank and lip of the caudal part of the sulcus principalis in area 9/46v and area 8Av (Figure 6, sections 1, 2). In the dorsolateral prefrontal cortex, label was observed in dorsal area 9/46 (i.e., area 9/46d) along the caudal half of the upper bank of the sulcus principalis and in area 8A. Terminal label was also observed in the dorsal premotor cortex in area 6DR.

#### Case 5

In this case, the isotope injection was placed in the ventral part of area PG just above and within the caudal-most part of the superior temporal sulcus (Figure 7). Labeled fibers were observed in the white matter of the inferior parietal lobule (Figure 7, section 5), which then coursed rostrally above the circular sulcus of the insula, i.e., in the location of the superior longitudinal fasciculus (Figure 7, section 4). In the ventrolateral frontal cortex, terminal label was observed in area 44 in the fundus of the inferior branch of the arcuate sulcus and in area 45B in the anterior bank of this sulcus (Figure 7, section 3). Further rostrally, terminal label was observed in area 45A, terminating just caudal to the IPD (Figure 7, section 2). Terminal label was also observed in the caudal half of the sulcus principalis involving area 9/46v. Fibers from the most ventral part of this injection were observed to arch as they entered the white matter of the inferior parietal lobule and thus forming part of the arcuate fasciculus.

#### Case 6

The isotope injection in this case was placed in the caudo-dorsal part of the inferior parietal lobule involving the caudal part of area PG and also area Opt, as well as the adjacent part of area POa in the intraparietal sulcus (Figure 8). Labeled fibers were observed in the white matter of the inferior parietal lobule and coursed rostrally above the circular sulcus of the lateral fissure in the superior longitudinal fasciculus (Figure 8, section 4). In the ventrolateral part of the frontal lobe, terminal label was observed primarily in area 45A (Figure 8, section 2). Some label was observed in area 45B (Figure 8, section 3). In the dorsolateral frontal cortex, terminal label was observed in areas 9/46v and 8Av.

### Lateral Superior Temporal Region

#### Case 7

In this case, the isotope injection was placed in the most rostral part of the superior temporal gyrus involving areas TS1 and TS2 (Figure 9). Labeled fibers were observed in the uncinate fasciculus and extreme capsule as they coursed towards the frontal lobe (Figure 9, section 5). The fibers running in the extreme capsule terminated within the ventrolateral frontal cortex in area 45B in the anterior bank of the inferior branch of the arcuate sulcus and in the rostral part of area 47/12 with only limited label in area 45A (Figure 9). The fibers running in the uncinate fasciculus terminated in the orbital part of area 47/12 and area 13 (Figure 9, sections 1–3).

#### Case 8

In this case, the isotope injection was placed in the parabelt region of the intermediate part of the superior temporal gyrus involving primarily area TS3 and encroaching slightly on the adjacent lip of the upper bank of the superior temporal sulcus (Figure 10). A large number of labeled fibers were observed in the immediately adjacent white matter of the superior temporal gyrus coursing anteriorly and dorsally in the extreme capsule (Figure 10, section 5). Terminal label was observed in the ventrolateral frontal cortex in area 44 in the depth of the inferior branch of the arcuate sulcus (Figure 10, section 3), in area 45B and in area 45A as far as the IPD (Figure 10, sections 1, 2). Terminal label was also observed in dorsolateral frontal areas 46, 9, and 8Ad, as well as in the frontopolar area 10.

#### Case 9

In this case the isotope injection was placed in the intermediate part of the superior temporal gyrus and involved primarily area paAlt and to a minor extent area TS3 (Figure 11). Labeled fibers were observed in the white matter of the superior temporal gyrus and these fibers coursed towards the frontal lobe in the extreme capsule (Figure 11, section 3). In the ventrolateral frontal cortex, terminal label was observed in area 45A and in the orbital part of area 47/12 (Figure 11, sections 1, 2). On the dorsolateral surface of the frontal lobe, terminal label was observed in areas 46, 9, 8Ad, and in frontopolar area 10.

#### Case 10

In this case, the isotope injection was placed in the upper bank of the superior temporal sulcus at the level of the central sulcus and involved mainly area TPO2 with some encroachment of the isotope on the lip of the superior temporal gyrus adjacent to the injection site (Figure 12). Labeled fibers were observed in the white matter of the superior temporal gyrus adjacent to the injection site and these fibers entered the extreme capsule as they coursed towards the frontal lobe (Figure 12, section 5). In the ventrolateral frontal cortex, terminal label was observed in area 45B and 45A as far as the IPD and more ventrally in area 47/12 (Figure 12, sections 1–4). In the dorsolateral frontal lobe, terminal label was observed in area 9 and in the frontopolar area 10. In the orbital surface of the frontal lobe, terminal label was observed in area 47/12 and in areas 13, 11, and in area 14 of the gyrus rectus. On the medial surface of the frontal cortex, there was some label in cingulate area 24.

#### Case 11

In this case, the isotope injection was placed in the dorsal part of area TE close to the lower bank of the superior temporal sulcus at the level of the central sulcus. The injection involved the dorsal portions of areas TE2 and TEm (Figure 13). Labeled fibers were observed in the white matter of the adjacent dorsal inferotemporal region and then these fibers coursed towards the frontal lobe in the extreme capsule (Figure 13, section 4). Within the ventrolateral frontal lobe, terminal label was observed in area 45A as far as the IPD (Figure 13, sections 1–3). Terminal label was also observed in area 45B and further dorsally close to the ventral bank of the caudal part of the sulcus principalis in area 9/46v.

## Discussion

**Figure 14:**
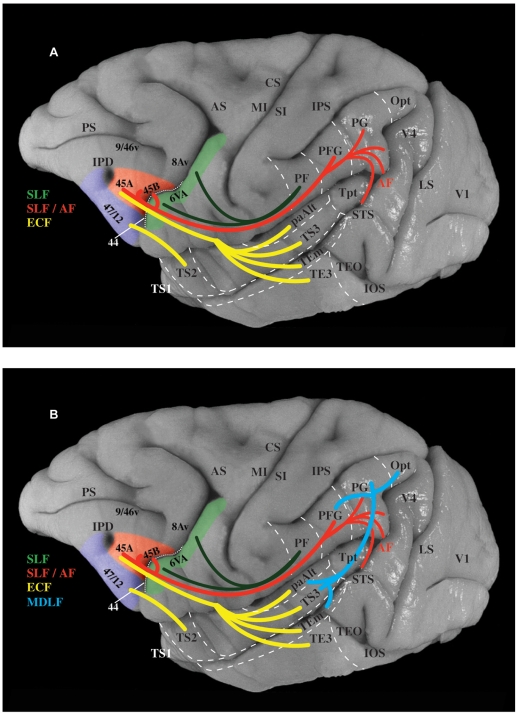
The dorsal and ventral pathways linking perisylvian regions with the homologues of Broca's region. (A) Photograph of the lateral surface of the macaque monkey brain onto which are projected the two pathways demonstrated in the present investigation. The ventral pathway (in yellow) is the extreme capsule fasciculus (ECF), which originates from cortical areas of the superolateral temporal region (i.e., superior temporal gyrus, superior temporal sulcus, and adjacent dorsal inferotemporal cortex), courses through the extreme capsule, and terminates primarily in area 45, with a more moderate projection to area 44. The dorsal pathway (in red) is the superior longitudinal fasciculus (SLF), which originates from areas of the inferior parietal lobule and terminates in areas 44, 45B, and 45A. Fibers originating from the caudal part of the superior temporal sulcus arch around the caudal end of the lateral fissure forming the arcuate fasciculus (AF) and blend with the fibers of the SLF in the white matter of the inferior parietal lobule. The ventral premotor cortex (area 6), which controls the orofacial musculature, receives strong input from the most rostral part of the inferior parietal lobule (area PF) via a part of SLF (shown in green). (B) Same as above, except that the massive system of fibers, the middle longitudinal fasciculus (MDLF), linking the superolateral temporal region with the inferior parietal lobule (previously demonstrated by Seltzer and Pandya [37],[40]) has been added to show that the suprasylvian inferior parietal lobule and the infrasylvian superolateral temporal regions, which are connected with Broca's region, are themselves massively interconnected. Thus, the lower panel shows the complete homologue of the circuitry that, in the left hemisphere of the human brain, will be used to serve linguistic processing when language develops. Note, however, that the circuitry already exists in the prelinguistic primate brain (see Discussion).

The present study of the parietal and temporal inputs to the ventrolateral frontal convexity of the macaque monkey demonstrated a number of fundamental facts about the connections of areas 44, 45B, and 45A, namely the homologues of Broca's region (Figures 1 and S1). The following facts were established with regard to parietal inputs: (a) Area 44 receives strong input from area PFG of the inferior parietal lobule, which corresponds to the caudal part of the supramarginal gyrus of the human brain. By contrast, the ventral premotor area 6, which controls the orofacial musculature and lies just caudal to area 44, receives most of its input from the most rostral part of the inferior parietal lobule (area PF), which corresponds to the most rostral part of the supramarginal gyrus in the human brain, and only a minor contribution from area PFG (compare Figures 3, 4, and 5). (b) Area 44 also receives input from the caudal half of the inferior parietal lobule, primarily from area PG, which corresponds to the cortex of the angular gyrus of the human brain. (c) Both subdivisions of area 45 (45B and 45A) receive parietal input from areas PFG and area PG. (d) The inputs from the inferior parietal lobule course via the second and third branches of the superior longitudinal fasciculus (SLF II and SLF III). These branches of the superior longitudinal fasciculus were first identified by Petrides and Pandya [16] but, at the time, their terminations in ventrolateral frontal cortex could not be identified as belonging to any of the subdivisions of Broca's region (areas 44, 45B, 45A), because these were only identified much later in the 1990s [8],[9],[15]. A contingent of axons originating from the most ventral part of area PG and the adjacent superior temporal sulcus forms an arch around the posterior end of the lateral fissure (the arcuate fasciculus), and these arching fibers then mingle with those of the superior longitudinal fasciculus as they course towards the ventrolateral frontal lobe. These superior longitudinal/arcuate axons form a *dorsal* stream of fibers that links the various areas of the inferior parietal lobule and adjacent superior temporal sulcus to the homologues of Broca's region in the frontal lobe (Figure 14).

In contrast to the above *dorsal* stream of axons that originate from the inferior parietal lobule and the adjacent most caudal parts of the superior temporal sulcus, the intermediate-to-anterior part of the superolateral temporal region sends axons that terminate in the ventrolateral frontal cortex via the extreme capsule (Figure S2B). We had originally demonstrated this *ventral* temporo-frontal pathway via the extreme capsule in the late 1980s [21], but we could not establish its precise terminations in the homologues of Broca's region because, at the time, these had not yet been recognized. The present study established the following new facts: (a) A very strong contingent of axons that originate in the intermediate and anterior parts of the superolateral temporal region and course in the extreme capsule terminate in area 45 (Figure 14), with a modest contingent of fibers terminating in area 44. (b) These temporo-frontal axons that form the extreme capsule fasciculus originate not only from the auditory superior temporal gyrus (Figures 9–11), but also from the multisensory cortex in the upper bank and depth of the superior temporal sulcus (Figure 12), and from the visual association cortex in the ventral bank of the superior temporal sulcus and the adjacent dorsal inferotemporal region (i.e., areas TEa/m and dorsal part of area TE) (Figure 13). (c) Axons running through the extreme capsule and directed to ventrolateral area 47/12 originate primarily from cortex in the ventral bank of the superior temporal sulcus and the adjacent inferotemporal area TE, while axons running via the uncinate fasciculus terminate in the orbital part of the frontal lobe including the orbital part of area 47/12.

In summary, the present study demonstrated that the long, afferent, monosynaptic, association axons that convey inferior parietal and lateral temporal inputs to the homologues of Broca's region are organized into a dorsal and a ventral stream of fibers (Figure 14). The dorsal stream can be subdivided into a rostral component of fibers via the third branch of the superior longitudinal fasciculus (SLF III) originating from the most rostral inferior parietal lobule and targeting the caudal ventrolateral frontal region (primarily rostral area 6 and area 44) and a caudal component originating from the intermediate-to-caudal inferior parietal lobule via the second branch of the superior longitudinal fasciculus (SLF II) and terminating in mid-ventrolateral areas 45A, 45B, and 44. The more ventral fibers of this system, originating from the parieto-temporal junction, arch around the end of the lateral fissure forming the arcuate fasciculus, which can therefore be thought of as the most ventral contingent of the superior longitudinal fibers. In the macaque monkey brain, the caudal inferior parietal lobule and adjacent caudal superior temporal sulcus exhibit a sharp upward direction (Figure 14). The ventral part of this region of the human brain occupies a much more ventral location (i.e., lies below the level of the end of the lateral fissure) because this parieto-temporal junction region has expanded pushing the lunate sulcus (i.e., the lateral border of the primary visual cortex) caudally as far as the occipital pole. Given the upward direction of the caudal inferior parietal lobule and adjacent caudal superior temporal sulcus in the macaque monkey, a significantly fewer number of fibers need to arch around the end-point of the lateral fissure in the monkey compared with the human brain. Thus, the arcuate fasciculus is not as prominent in the monkey [39] although, as shown here, undoubtedly present and a part of the dorsal stream of axons.

The ventral temporo-frontal stream of fibers originates from the intermediate and anterior parts of the lateral temporal cortex and targets primarily area 45 and to a modest extent area 44. These fibers form the extreme capsule fasciculus (Figures 14 and S2B) [21]. It should be pointed out here that the inferior parietal lobule and adjacent caudal superior temporal sulcus (origins of the dorsal stream) and the intermediate-to-anterior superolateral temporal region (origin of the ventral stream) are known to be massively interconnected via the middle longitudinal fasciculus (Figure 14), which was first established in the macaque monkey by Seltzer and Pandya [37],[40] and more recently demonstrated in the human brain with diffusion tensor imaging [41].

The findings established in the present investigation are of major significance for understanding the precursor neural circuitry in the nonlinguistic nonhuman primate brain, which, in the left dominant hemisphere of the human brain, was adapted to serve language processing. These findings naturally raise the question of the extent to which the precursor neural circuitry is still present in the human brain. The traditional view of human language circuitry has been that the caudal temporal region (Wernicke's region) is connected with the anterior language zone (Broca's region) via the arcuate fasciculus and this view has dominated theoretical attempts to interpret language processing and its disorders (e.g., [42]). The present findings show that, even in the macaque monkey, there is a much richer system of pathways linking posterior parietal and lateral temporal cortex with the ventrolateral frontal region than the traditional view would suggest. As pointed out above, although diffusion tensor imaging in the human brain does not have the resolution to establish the origins and precise terminations of such pathways in the human brain, it has provided evidence that a rich system of comparable pathways also exist in the human brain (e.g., [27]–[31]). For instance, in a recent diffusion tensor imaging study in the human brain [31], we were able to establish a pathway running from the superolateral temporal region via the extreme capsule towards the ventrolateral frontal region and another pathway running from the rostral inferior parietal lobule via the superior longitudinal fasciculus towards this same region, consistent with the present findings in the macaque monkey and also suggestions from earlier diffusion tensor imaging work (e.g., [27]–[30]). On the basis of the present experimental anatomical results in the monkey that provide details of origins and precise terminations of these pathways, we can assume that the target of the extreme capsule fibers from the superolateral temporal cortex and the superior longitudinal fasciculus from the inferior parietal lobule is indeed Broca's region.

### Functional and Evolutionary Considerations

Several functional neuroimaging studies that searched for the articulatory/phonological system of the human brain observed coactivation of area 44 (i.e., the articulatory part of Broca's region) with the rostral part of the inferior parietal lobule, namely the supramarginal gyrus [43],[44]. It is therefore of considerable interest that the present experimental anatomical results in the macaque monkey demonstrate that the rostral part of the inferior parietal lobule (areas PF and PFG that correspond to the human supramarginal gyrus) is strongly linked with area 44 and the ventral part of the premotor cortex (area 6), which controls the orofacial musculature, and that these connections are made via a distinct branch of the superior longitudinal fasciculus (SLF III). Electrophysiological studies in the macaque monkey have shown that the rostral part of the inferior parietal cortex is involved with hand and orofacial action control (e.g., [45]–[48]). Thus, the specific part of the inferior parietal lobule that is linked with ventral premotor area 6 and area 44 plays a role in orofacial and hand action control. Furthermore, a number of investigators of human language have recently suggested that there is a dorsal stream involved in the mapping of sound-to-articulation and a ventral stream for mapping sound-to-meaning (e.g., [49],[50]). A study that combined functional magnetic resonance imaging with diffusion tensor imaging has provided evidence that sublexical repetition of speech, which requires sound-to-articulation transformations, involves a dorsal system that includes the superior longitudinal/arcuate fasciculus and the premotor cortical area 6 and area 44 [44]. In other words, there is now evidence that the dorsal stream that we demonstrated here in the macaque monkey and have shown to terminate strongly in premotor area 6 and area 44 has been adapted, in the human brain, for use in sound-to-speech articulation transformations. In addition, in the human brain, the dorsal system with its linkages to posterior temporal cortex is involved in syntactic processing (e.g., [51]). Clearly, these are functional contributions of these systems characteristic of the human brain and have possibly evolved as a result of the considerable expansion of the parieto-temporal region in the human brain, including the associated Broca's region in the ventrolateral frontal cortex. There is also evidence that sound-to-meaning comprehension involves primarily the ventral stream of fibers connecting the intermediate lateral temporal cortex with the ventrolateral region via the extreme capsule [44].

What is the role of Broca's homologues in the macaque monkey? Before addressing this question, we should note that architectonic areas 44 and 45 (Broca's homologues) exist also in the ventrolateral frontal region of the human nondominant right hemisphere, namely the hemisphere that is heavily involved in nonlinguistic spatial and nonspatial processing. We should therefore ask: What might be the general nonlinguistic role of the ventrolateral frontal cortical region in the human nondominant right hemisphere and in the macaque monkey? Petrides [52] has argued that a fundamental contribution of nonlinguistic ventrolateral prefrontal areas 45 and 47/12 is the active controlled retrieval of information from memory, namely controlled retrieval of mnemonic information stored in posterior cortical association areas. In functional magnetic resonance imaging studies, we were indeed able to show that the human ventrolateral prefrontal cortex in the *right* hemisphere is critically involved in the active controlled retrieval of *nonverbal* visual information, such as information about abstract designs, faces, and locations [53],[54]. We then used the exact same nonverbal active controlled retrieval paradigm in macaque monkeys and have shown that single neuron activity in areas 45 and 47/12 of the nonhuman primate brain is involved with the active controlled retrieval of visual object and spatial information [55]. Thus, ventrolateral prefrontal cortex in the macaque monkey, as in the right hemisphere of the human brain, plays a major role in the controlled strategic retrieval of nonlinguistic information from memory. We can therefore suggest that this nonlinguistic contribution of prefrontal areas 45 and 47/12 was adapted, in the left hemisphere of the human brain, to serve controlled retrieval of verbal information [56], just as the hippocampal region in the left hemisphere of the human brain has been adapted to serve verbal declarative memory encoding while in nonhuman animals and the human right hemisphere supports nonverbal spatial declarative memory [57],[58]. Indeed, there is now considerable evidence from many laboratories that controlled, strategic, verbal memory retrieval and selection involves left ventrolateral prefrontal cortex, area 45, and the related area 47/12 (e.g., [59]–[62]).

In summary, the evidence reviewed above has suggested that one of the component areas of Broca's region, namely area 45 and the related ventrolateral area 47/12, is involved with the controlled retrieval and selection of information from nonverbal memory in the monkey and in the right nondominant hemisphere of the human brain. How might the other component of Broca's region, namely area 44, be related to function? Area 44 is located between area 45, on the one hand, and ventral premotor cortex (area 6) controlling hand/arm and oral/facial musculature, on the other hand, and is connected with both these adjacent areas. Thus, area 44 is in an ideal position to mediate between strategically retrieved and selected information from posterior temporal and parietal cortex by area 45 and the articulation of such information via hand and orofacial action by the ventral precentral gyrus (i.e., the premotor and motor cortex) (see Petrides [56]). In other words, these results suggest that area 44 might be a ventrolateral frontal control area involved in the highest levels of programming of action that will be instantiated by a series of articulatory acts regulated by the premotor and motor cortex of the precentral gyrus. During the evolution of the human brain, these high-level forms of programming (the basic elements of which are already present in the macaque monkey brain) came to include complex syntactical structure (e.g., hierarchical level of control) that is necessary for language (in the narrow sense), and which has been argued to be a major contribution of Broca's region (e.g., [63],[64]). If we were to extrapolate these arguments on the basis of the present monkey anatomical study, our recording study in the monkey [55], and our functional neuroimaging studies of the human right hemisphere homologue of Broca's region [53],[54], we could say that a common primate circuitry was adapted, during millions of years of evolution, in the human brain for the strategic retrieval and selection of information from verbal memory (including the mental lexicon) in posterior temporo-parietal cortical regions by one component of Broca's region, area 45, and the transformation of this selected conceptual information into gestural/speech acts by the other component of Broca's region, area 44, via its connections with motor structures, such as the premotor cortex, the basal ganglia, and the rostral inferior parietal lobule (see Petrides [56] for this argument). We should emphasize here that, clearly, macaque monkeys do not have complex syntactic processing. Our suggestion here is simply that an area that served higher control of action in the macaque monkey may have been adapted for the control of complex hierarchical sequences of gestural and vocal action with the evolution of communication leading to human speech.

When might this adaptation have begun? There is paleoneurological evidence from fossil endocasts that the asymmetry in Broca's region observed in the modern human brain can be observed in brain endocasts of specimens assigned to *Homo erectus/habilis*, based on the petalia impressions [65] and the study of the endocast of the Sambungmacan 3 (Sm3) fossil, a *Homo erectus* calvaria from Indonesia [66]. For instance, Sm3 exhibits left-right cerebral volume asymmetry and marked asymmetry in Broca's cap, i.e., modern human characteristics. Thus, the fossil evidence suggests that this asymmetry is a relatively recent event in the evolution of the human brain. Although the presence of this asymmetry does not necessitate the conclusion that there was modern language lateralization as early as *Homo erectus*, it can be interpreted as supportive evidence that some precursor of the modern function of Broca's region may have begun evolving in *Homo erectus*. Although language in the narrow sense is clearly a human characteristic, it is interesting to note the presence of areas 44 and 45 (Broca's region) in the brain of the African great apes [67] and also the presence of various asymmetries in the chimpanzee brain relevant to our understanding of the evolution of language, such as a leftward asymmetry in the planum temporale based on architecture [68] and in the ventrolateral frontal region based on gross morphological features [69]. More recently, behavioral evidence has been presented for left hemisphere dominance in the chimpanzee brain for the control of oro-facial movements associated with learned communicative vocalizations, while emotional stereotyped vocalizations may be controlled by the right hemisphere [70]. In addition, in chimpanzees, handedness for tool use has been linked to asymmetries in the ventrolateral frontal region and the planum temporale [71], suggesting a left hemisphere specialization for the control of complex action using the right hand, paralleling a well-known phenomenon in the human brain [72]. These findings are consistent with suggestions that specialization for the control of action and gesture may have preceded specialization for language (e.g., [72]–[74]). Note also that our close primate relatives, chimpanzees and bonobos, use arm/hand gestures more flexibly in their natural communication across contexts than facial expressions and vocalizations [75]. The above facts suggest that the use of gestures for early forms of communication may have been an adaptation distinguishing the Hominoidea from other primates, and that the use of vocalization in the form of modern speech emerged much later with the evolution of language in the narrow sense, i.e., a uniquely human adaptation. It is interesting in this respect that the supralaryngeal vocal tract of humans differs significantly from those of other primates, making the human vocal apparatus unique in transmitting information at fast rates [76].

In conclusion, the present findings indicate that, even in the nonlinguistic nonhuman primate brain, the precursors of Broca's region have a wide neural circuitry involving specific connections with posterior inferior parietal cortex and adjacent caudal superior temporal sulcus and with the intermediate-to-anterior superolateral temporal cortex. It is likely therefore that these pathways, which are additional to the posterior temporal-to-frontal connection via the arcuate fasciculus (traditionally assumed to be the main language pathway), play a major role in language processing in the human brain. The present anatomical findings indicate a rich dorsal stream of fibers via the superior longitudinal/arcuate fasciculus that can be further subdivided into a rostral and a caudal component and an independent ventral temporo-frontal system via the extreme capsule fasciculus that targets predominantly the prefrontal cortical areas 45 and 47/12. Recent diffusion tensor imaging studies (e.g., [27]–[31]) indicate the existence of similar systems in the human brain. This fact suggests that the circuitry of Broca's homologues demonstrated in the present macaque monkey study, which provides details about cortical origins and terminations of pathways not available in the human brain, is most probably also true (and even further elaborated) in the human brain. These findings indicate rich functional interactions between posterior perisylvian cortical regions and the subdivisions of Broca's region, i.e., areas 44, 45B, and 45A, via pathways other than those made by the traditional arcuate fasciculus and raise hypotheses to be tested in the human brain by combined functional magnetic resonance imaging and diffusion tensor imaging. Furthermore, and equally important, it opens up the possibility of examining the neurophysiological basis of the prelinguistic use of these pathways (e.g., [55]), a use that is still directly relevant to the function of these areas in the nondominant right hemisphere of the human brain (e.g., [53],[54]), and thus provide major insights into neural computations that were adapted to serve language processing with the emerging specialization of the human left hemisphere for language processing. In this manner, the study of the functional contribution of the nonlinguistic macaque monkey circuitry of Broca's homologues can potentially provide important insights into the evolution of language.

## Materials and Methods

### Surgery

Injections of radioactively labeled amino acids were placed in different parts of the posterior parietal cortex and the temporal cortex in 11 rhesus monkeys (*Macaca mulatta*). The injections were placed in the left hemisphere (cases 1, 3, 4, 5, 6, 8, 9, 11) (Figures 3,5–8,10,11, and 13), except for three cases in which they were placed in the right hemisphere (cases 2, 7, 10) (Figures 4, 9, and 12). In the latter three cases (Figures 4, 9, and 12), the drawings were left/right reversed so that the orientation would be consistent with that in all other figures and help the reader in comparing cases across figures. The care and use of animals were in accordance with the guidelines of the National Institutes of Health and the Canadian Council for Animal Care. The animals were immobilized with ketamine hydrochloride (10 mg/kg) and then deeply anesthetized with sodium pentobarbital (30 mg/kg) (Sigma) administered intravenously. A craniotomy was then performed, under aseptic surgical techniques, over the region of interest. In each case, an attempt was made to place two juxtaposed isotope injections into an architectonic area in the parietal or the temporal cortex. The intracortical injections consisted of radioactively labeled amino acids (^3^H-leucine and/or proline; volume range, 0.4–1.0 µl; specific activity range, 40–80 µCu, aqueous solution; New England Nuclear Brand Radiochemicals from PerkinElmer). After survival periods ranging from 7 to 10 d, the animals were deeply anesthetized with sodium pentobarbital and perfused transcardially with physiologic saline, followed by a 10% formalin solution.

### Tissue Processing and Microscopic Examination

The brains were divided into two blocks by a coronal cut and photographed from all angles. They were subsequently embedded in paraffin, sectioned at 16-µm thickness, and processed for autoradiography according to the technique described by Cowan et al. [32]. The exposure times varied between 3 and 6 mo. At monthly intervals, trial sections were developed for identification of optimal radiolabeling. The sections were also counterstained with thionine to permit identification of the architectonic areas. A series of coronal sections of the hemisphere were examined microscopically with darkfield illumination (for examples see Figures S2 and S3). The labeled fibers in the white matter and the terminal labeling in the cerebral cortex and subcortical structures were recorded with the aid of an X-Y plotter (Hewlett Packard) that was electronically coupled to the stage of the microscope (Leitz Aristoplan). This information was then used to reconstruct the injection and termination sites, as well as the path of the labeled fibers. The cytoarchitectonic boundaries of the projection areas within the cerebral cortex, as well as the sites of origin of the pathways were established in the experimental material under light field illumination.

The distribution of the terminations of the labeled fibers was transferred onto 2-D reconstructions of the lateral, medial, and ventral surfaces of the examined cerebral hemispheres. These 2-D reconstructions of the hemispheres were made using a precision technical drawing software program (AutoSketch, Release 7, Autodesk, Inc.). In order to minimize the distortion in the normal view of the cerebral hemisphere, which inevitably follows such maps, we unfolded the cortex lying within the major sulci separately. These unfolded sulci are presented next to the lateral views of the hemispheres so that the reader can appreciate the details of the terminations within the sulci. On the coronal sections of the hemisphere that was to be reconstructed, we traced the distance from the midline (i.e., the border of the lateral with the medial surface of the hemisphere) to the first sulcus encountered laterally. We then measured the distance from that sulcus to the next sulcus and so on until the lateral-to-ventral edge of the hemisphere was reached. These measurements were used for the lateral surface reconstruction. For the reconstruction of the medial surface of the frontal lobe, we measured the distance from the dorsal-most part of the midline to the first sulcus encountered ventrally and then to the next sulcus until the ventral-most part of the medial surface was reached. The orbital surface of the frontal lobe was reconstructed by measuring the distance, on each coronal section, from the midline to the first sulcus encountered laterally (i.e., the medial orbital sulcus) and then from there to the lateral orbital sulcus and then to the ventral-to-lateral edge of the hemisphere. The medial/ventral surface of the temporal lobe was included with the reconstruction of the medial surface of the hemisphere and the origin of the measurements was the hippocampal sulcus. The measurements obtained for the lateral, medial, and orbital surfaces, as well as the cortex within the sulci, were thus a series of line segments (the *y* coordinates) arranged in the anteroposterior direction (*x* coordinates). In separate spreadsheets, the points (*x*, *y* coordinates) were plotted and joined together in order to reconstruct the 2-D flattened outlines of surfaces of the hemisphere and the sulci.

## Supporting Information

Figure S1 **Light field photomicrographs of cortical area 44 and area 45B.** The part of each photomicrograph lying between the two horizontal lines is expanded (on the right side of the figure) to show details of the lower part of layer III, layer IV, and layer V. Note the well-developed granular layer IV in area 45 and the clusters of large and deeply stained neurons in the deep part of layer III. Thus, area 45 is a clearly granular cortical area. By contrast, layer IV in area 44 is narrow and interrupted, which leads to the description of area 44 as “dysgranular” cortex. Calibration bar, 1 mm.
(4.68 MB TIF)

Figure S2 **Photomicrographs.** (A) Photomicrograph of a small part of the ventrolateral prefrontal cortex in case 8 (see Figure 10, section 1, inset) to show terminal label in cortex. The inset shows the deep part of layer III and layers IV and V of this small patch of cortex. The inset is expanded on the right side and shown in light field to demonstrate that the terminal label is in a part of ventrolateral prefrontal cortex that has large neurons in deep layer III and a well developed layer IV, i.e., in area 45A. (B) Photomicrograph of the injection site in case 10 (in the upper bank of the superior temporal sulcus and adjacent white matter) to show the origin of the extreme capsule fasciculus (ECF) as it courses dorsally between the insula (IN) and the claustrum (CL) to enter the extreme capsule and course towards the frontal lobe. This arrangement was typical of all cases with temporal lobe injections that demonstrated the ECF. Note that another branch of labeled fibers from the injection site is directed ventrally to other parts of the temporal lobe and a central branch is directed medially towards the thalamus and other medially located structures. Each one of the rectangles that constitute the overall photomicrograph is 2,955 µm by 2,205 µm and were taken by means of a motorized XY microscope stage and a computer running Stereo Investigator software (Microbrightfield, Inc.). Abbreviations: CL, claustrum; E, external capsule; ECF, extreme capsule fasciculus; IN, insula; LF, lateral fissure; LV, lateral ventricle; Pu, putamen; STS, superior temporal sulcus.
(7.98 MB TIF)

Figure S3 **Darkfield photomicrographs of injection sites.** (A) Injection site in the dorsal part of the inferior parietal lobule (area PG) in case 4. Notice the labeled band of fibers that are entering the white matter of the inferior parietal lobule to form the superior longitudinal fasciculus. We can also see in this section local U-fibers that are entering in columns the more ventral parts of area PG to form intra-areal connections. (B) Injection site in superior temporal gyrus (area paAlt) in case 9. Note the dorsomedially directed bundle of fibers that is going to enter the extreme capsule on its way to the frontal lobe. Abbreviations: IPS, intraparietal sulcus; LF, lateral fissure; STS, superior temporal sulcus.
(6.10 MB TIF)

## Abbreviations

IPD: infraprincipal dimple
