# Supplementary figures and images for: Distinct Parietal and Temporal Pathways to the Homologues of Broca's Area in the Monkey
Source: PLoS Biol. 2009 Aug 11;7(8):e1000170. doi: 10.1371/journal.pbio.1000170 (PMC2714989; doi:10.1371/journal.pbio.1000170)

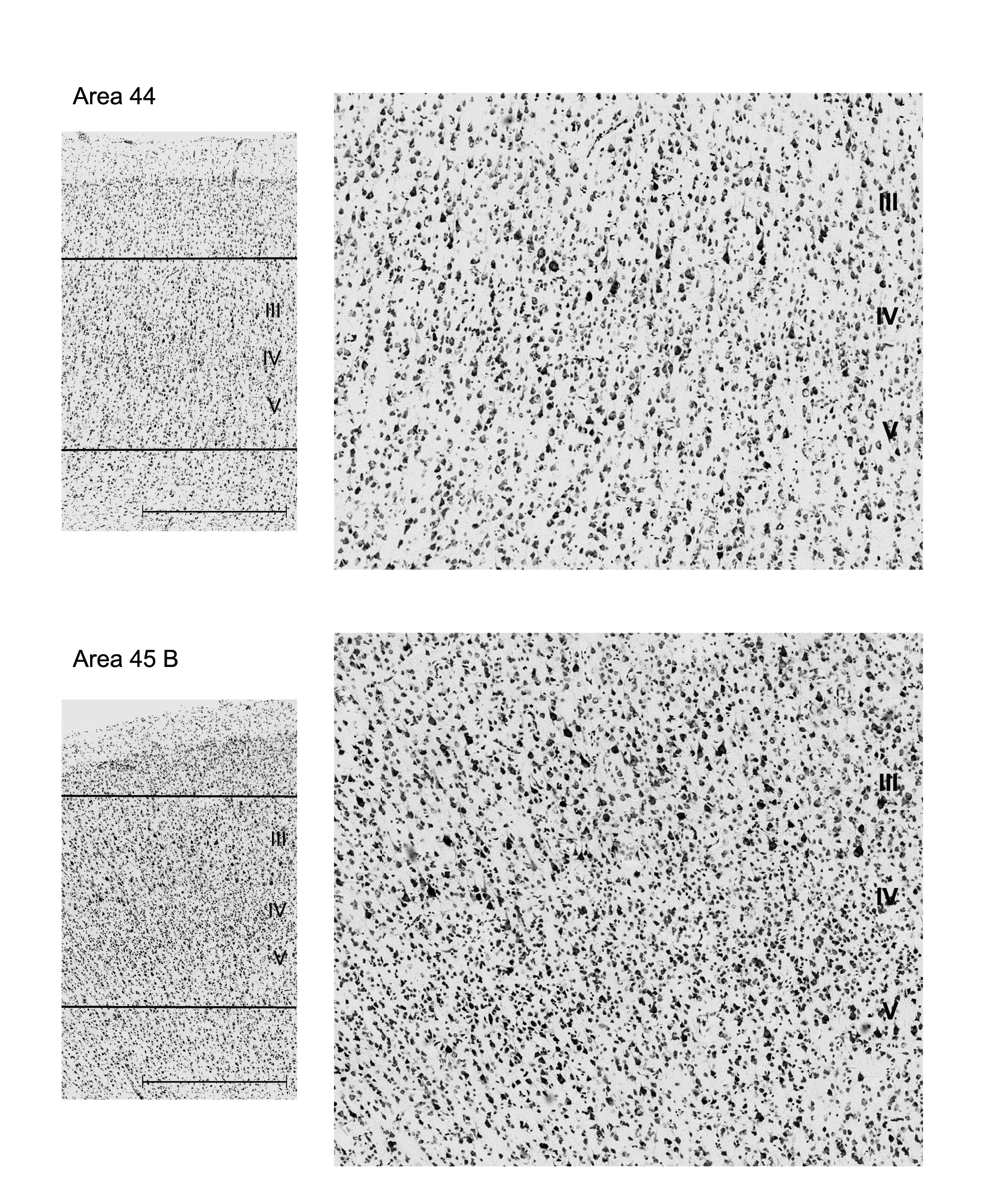

Supplement: Figure S1 — Light field photomicrographs of cortical area 44 and area 45B. The part of each photomicrograph lying between the two horizontal lines is expanded (on the right side of the figure) to show details of the lower part of layer III, layer IV, and layer V. Note the well-developed granular layer IV in area 45 and the clusters of large and deeply stained neurons in the deep part of layer III. Thus, area 45 is a clearly granular cortical area. By contrast, layer IV in area 44 is narrow and interrupted, which leads to the description of area 44 as “dysgranular” cortex. Calibration bar, 1 mm. (4.68 MB TIF) [file pbio.1000170.s001.tif]

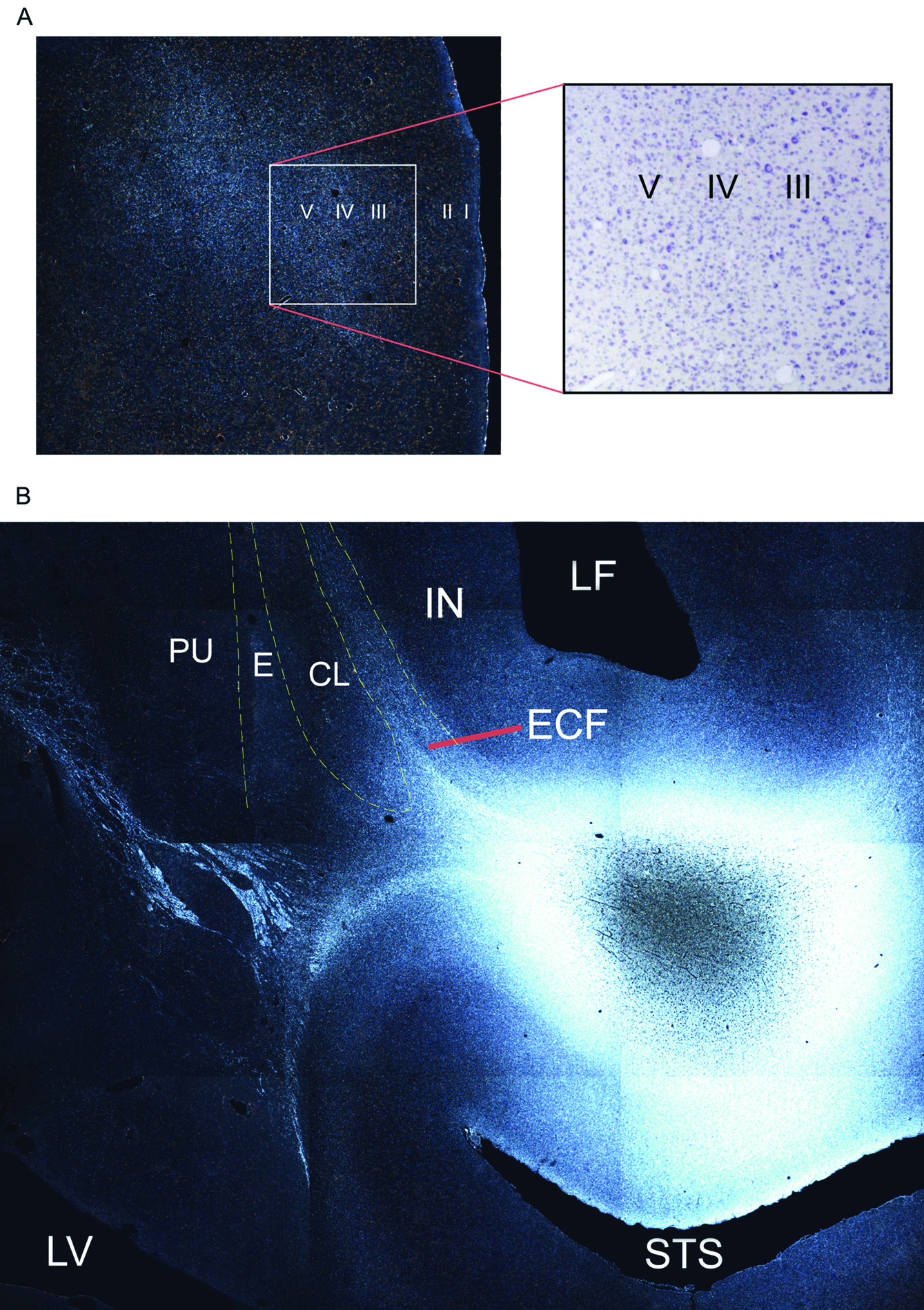

Supplement: Figure S2 — Photomicrographs. (A) Photomicrograph of a small part of the ventrolateral prefrontal cortex in case 8 (see Figure 10, section 1, inset) to show terminal label in cortex. The inset shows the deep part of layer III and layers IV and V of this small patch of cortex. The inset is expanded on the right side and shown in light field to demonstrate that the terminal label is in a part of ventrolateral prefrontal cortex that has large neurons in deep layer III and a well developed layer IV, i.e., in area 45A. (B) Photomicrograph of the injection site in case 10 (in the upper bank of the superior temporal sulcus and adjacent white matter) to show the origin of the extreme capsule fasciculus (ECF) as it courses dorsally between the insula (IN) and the claustrum (CL) to enter the extreme capsule and course towards the frontal lobe. This arrangement was typical of all cases with temporal lobe injections that demonstrated the ECF. Note that another branch of labeled fibers from the injection site is directed ventrally to other parts of the temporal lobe and a central branch is directed medially towards the thalamus and other medially located structures. Each one of the rectangles that constitute the overall photomicrograph is 2,955 µm by 2,205 µm and were taken by means of a motorized XY microscope stage and a computer running Stereo Investigator software (Microbrightfield, Inc.). Abbreviations: CL, claustrum; E, external capsule; ECF, extreme capsule fasciculus; IN, insula; LF, lateral fissure; LV, lateral ventricle; Pu, putamen; STS, superior temporal sulcus. (7.98 MB TIF) [file pbio.1000170.s002.tif]

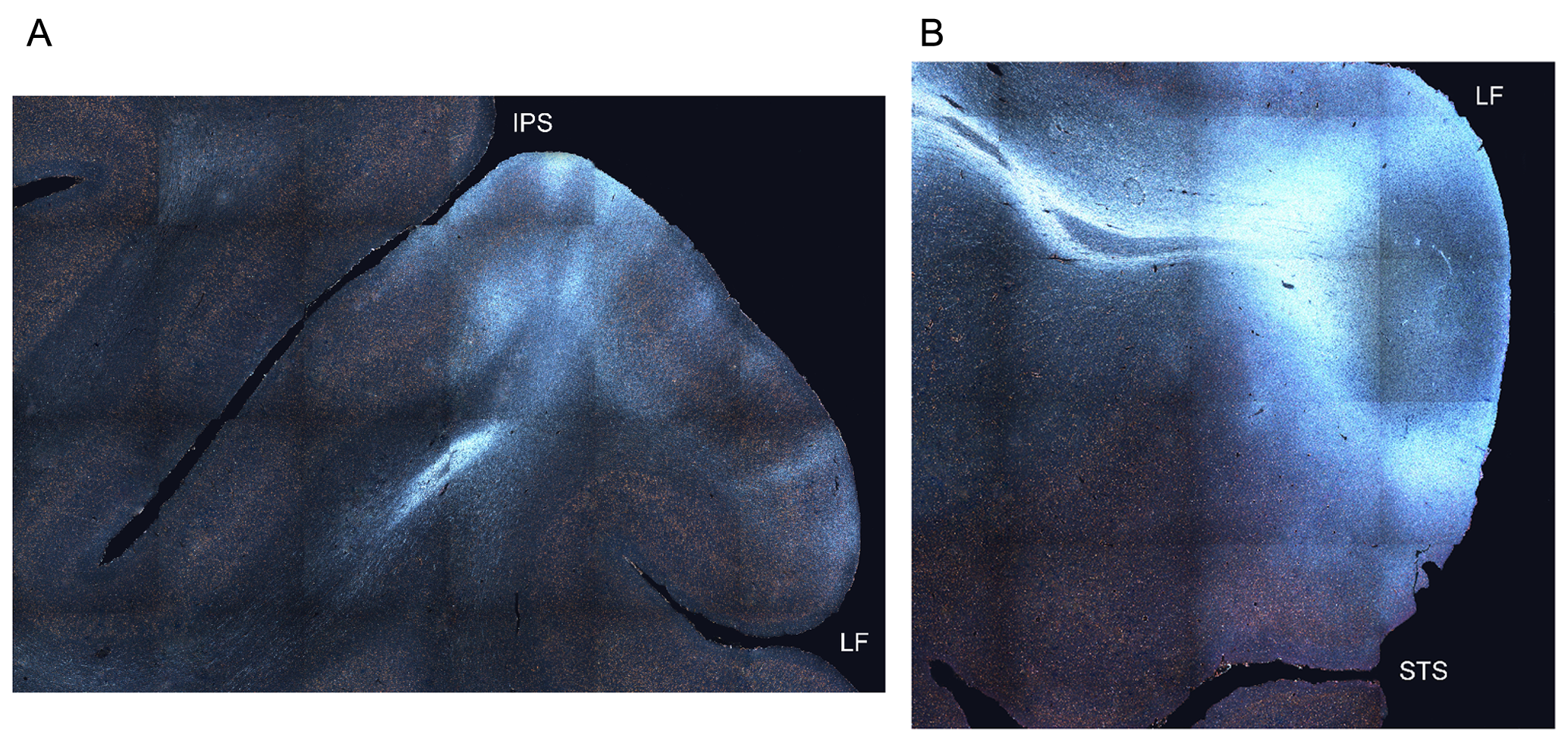

Supplement: Figure S3 — Darkfield photomicrographs of injection sites. (A) Injection site in the dorsal part of the inferior parietal lobule (area PG) in case 4. Notice the labeled band of fibers that are entering the white matter of the inferior parietal lobule to form the superior longitudinal fasciculus. We can also see in this section local U-fibers that are entering in columns the more ventral parts of area PG to form intra-areal connections. (B) Injection site in superior temporal gyrus (area paAlt) in case 9. Note the dorsomedially directed bundle of fibers that is going to enter the extreme capsule on its way to the frontal lobe. Abbreviations: IPS, intraparietal sulcus; LF, lateral fissure; STS, superior temporal sulcus. (6.10 MB TIF) [file pbio.1000170.s003.tif]
